# 2-({1-[2-(Methyl­sulfan­yl)phen­yl]-1*H*-tetra­zol-5-yl}sulfan­yl)acetic acid

**DOI:** 10.1107/S160053681300980X

**Published:** 2013-04-20

**Authors:** Ana C. Mafud, Yvonne P. Mascarenhas, Alessandro S. Nascimento

**Affiliations:** a Instituto de Física de São Carlos, Av. do Trab. Sãocarlense, 400, São Carlos, SP, Brazil

## Abstract

In the title compound, C_10_H_10_N_4_O_2_S_2_, the tetra­zole and benzene rings are almost normal to one another, with a dihedral angle between their planes of 84.33 (9)°. In the crystal, mol­ecules are linked *via* pairs of bifurcated O—H⋯(N,N) hydrogen bonds, forming inversion dimers with graph-set motif *R*_4_^4^(12). The dimers are linked by significant π–π inter­actions involving inversion-related tetra­zole rings and inversion-related benzene rings, with centroid–centroid distances of 3.7376 (14) and 3.8444 (15) Å, respectively.

## Related literature

For details of the ZINC database, see: Irwin *et al.* (2012 ▶). For information on the biological properties of tetra­zoles, see: Kees *et al.* (1989 ▶); Nolte *et al.* (1998 ▶); Mafud & Nascimento (2013 ▶).
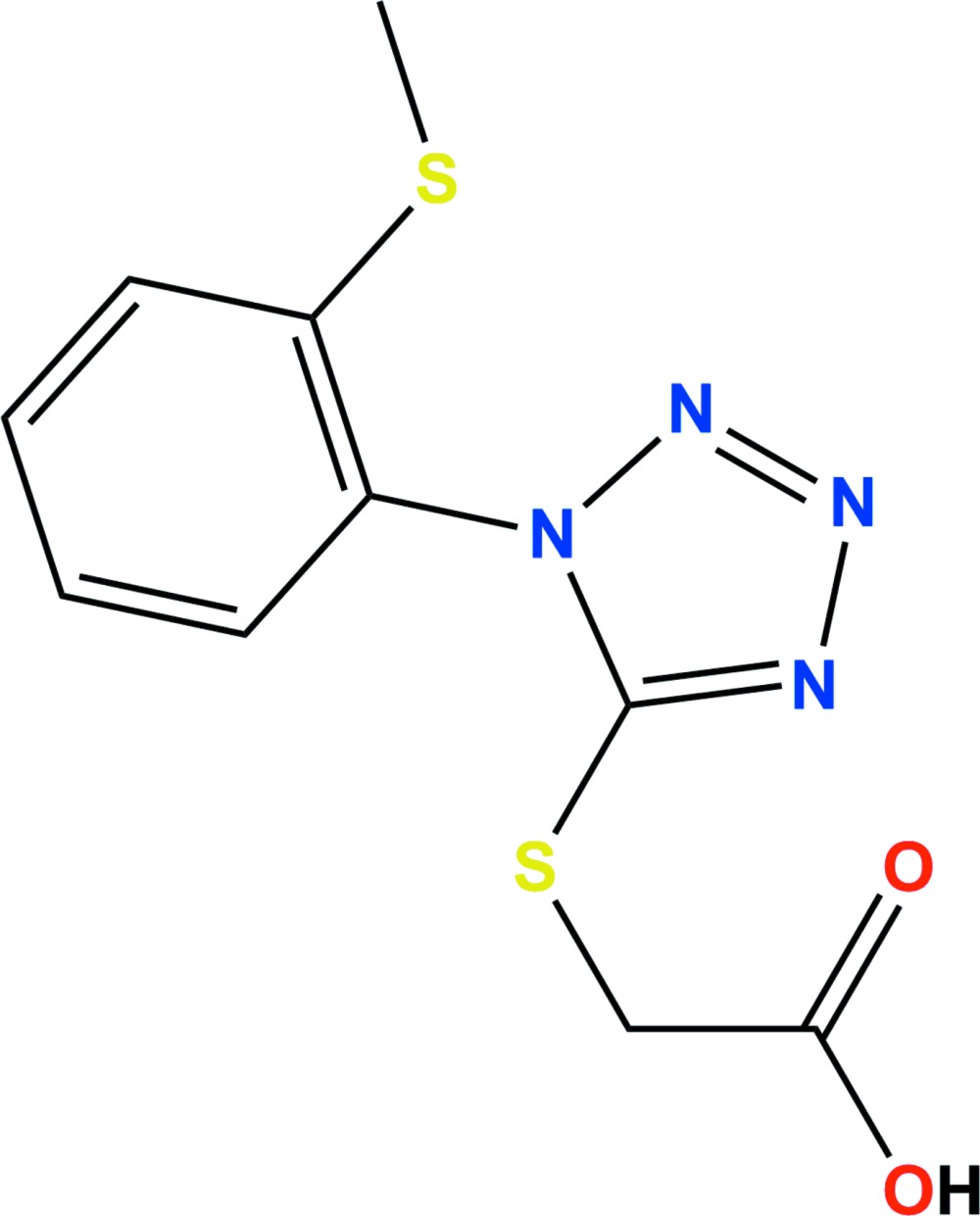


## Experimental

### 

#### Crystal data

C_10_H_10_N_4_O_2_S_2_
*M* *_r_* = 282.34
Triclinic, 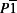

*a* = 7.1500 (3) Å
*b* = 8.3770 (3) Å
*c* = 11.0890 (5) Å
α = 74.7480 (14)°
β = 79.3090 (14)°
γ = 86.286 (3)°
*V* = 629.58 (4) Å^3^
*Z* = 2
Mo *K*α radiation
μ = 0.42 mm^−1^
*T* = 290 K
0.1 × 0.05 × 0.05 mm

#### Data collection

Bruker–Nonius KappaCCD diffractometer
Absorption correction: for a cylinder mounted on the ϕ axis (Dwiggins, 1975 ▶) *T*_min_ = 0.861, *T*_max_ = 0.862
15888 measured reflections
2335 independent reflections
1879 reflections with *I* > 2σ(*I*)
*R*_int_ = 0.079

#### Refinement

*R*[*F* ^2^ > 2σ(*F* ^2^)] = 0.051
*wR*(*F* ^2^) = 0.15
*S* = 1.04
2335 reflections
167 parameters
H atoms treated by a mixture of independent and constrained refinement
Δρ_max_ = 0.50 e Å^−3^
Δρ_min_ = −0.30 e Å^−3^

### 

Data collection: *COLLECT* (Nonius, 1999 ▶); cell refinement: *SCALEPACK* (Otwinowski & Minor, 1997 ▶); data reduction: *DENZO* (Otwinowski & Minor, 1997 ▶) and *SCALEPACK*; program(s) used to solve structure: *SIR92* (Altomare *et al.*, 1994 ▶); program(s) used to refine structure: *SHELXL97* (Sheldrick, 2008 ▶); molecular graphics: *ORTEP-3 for Windows* (Farrugia, 2012 ▶) and *Mercury* (Macrae *et al.*, 2008 ▶); software used to prepare material for publication: *WinGX* (Farrugia, 2012 ▶).

## Supplementary Material

Crystal structure: contains datablock(s) global, I. DOI: 10.1107/S160053681300980X/su2571sup1.cif

Structure factors: contains datablock(s) I. DOI: 10.1107/S160053681300980X/su2571Isup2.hkl

Supplementary material file. DOI: 10.1107/S160053681300980X/su2571Isup3.cml

Additional supplementary materials:  crystallographic information; 3D view; checkCIF report

## Figures and Tables

**Table 1:** Hydrogen-bond geometry (Å, °)

*D*—H⋯*A*	*D*—H	H⋯*A*	*D*⋯*A*	*D*—H⋯*A*
O1—H1⋯N1^i^	0.81 (4)	2.15 (4)	2.952 (4)	176 (4)
O1—H1⋯N2^i^	0.81 (4)	2.51 (4)	3.232 (4)	149 (4)

Symmetry code: (i) 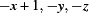.

**Fig. 1.**
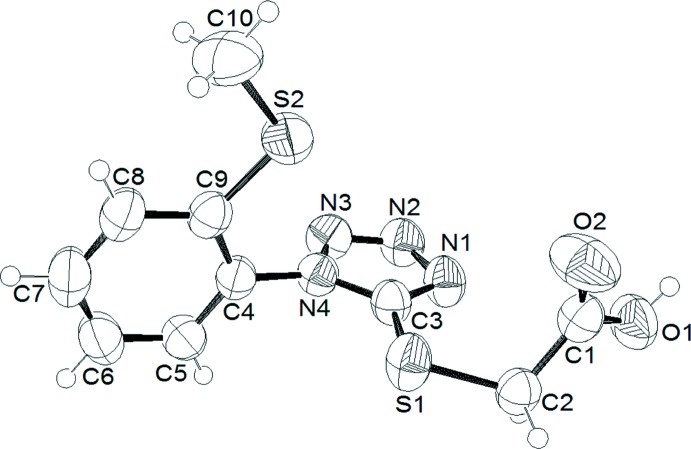
A view of the molecular structure of the title molecule, with atom labelling. The displacement ellipsoids are drawn at the 50% probability level.

**Fig. 2.**
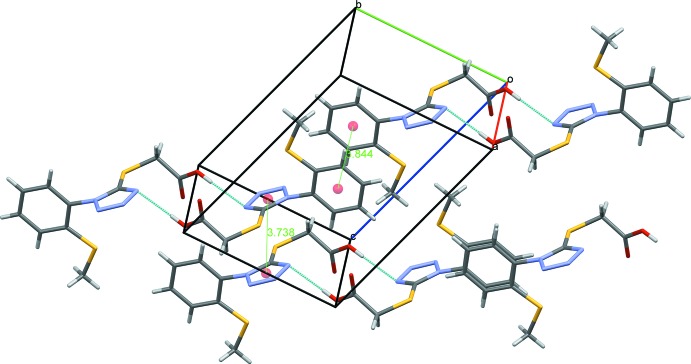
A view of the crystal packing of the title compound, illustrating the O—H···N hydrogen bonds (dashed lines; see Table 1 for details) and the π-π interactions (red ball = ring centroid).

## supplementary crystallographic information

### Comment

The title acid is a screening molecule available in the ZINC database (Irwin *et al.*, 2012) among the 'drugs-now' subset. This molecule has been identified as a PPAR gamma ligand candidate in a virtual screening study. The peroxisome proliferator-activated receptors, isoform gamma, are a transcription factors whom regulating the genes expression (Nolte *et al.*, 1998). The binding was further confirmed in experimental binding assays (Mafud *et al.*, 2013). Since tetrazoles are already known to have glucose lowering effects *in vivo* (Kees *et al.*, 1989), in this virtual screening we chose some different representative molecules to evaluate the affinities and the extent of receptor activation. We report herein on the crystal structure of the title compound.

The molecular structure of the title molecule is illustrated in Fig. 1. The tetrazole and phenyl rings are almost normal to one another with a dihedral angle of 84.33 (9)°.

In the crystal, molecules are linked *via* O—H···N hydrogen bonds forming inversion dimers with graph-set motif *R*^4^_4_(12); see Fig. 2 and Table 1. The dimers is linked by significant π–π interactions involving inversion related tetrazole rings (*Cg*1 centroid of ring N1—N4/C3) and inversion related phenyl rings (*Cg*2 centroid of ring C4—C9): *Cg*1···*Cg*1^i^ = 3.7376 (14) Å; *Cg*2···*Cg*2^ii^ = 3.8444 (15) Å; symmetry codes: (i) -*x+1*, -*y+1*, -*z*; (ii) -*x+1*, -*y+1*, -*z+1*.

### Experimental

A yellow prism-like crystal of the title compound was selected from the sample as supplied (ChemBridge Corporation) without recrystallization.

### Refinement

The hydroxyl H atom was located in a difference Fourier map and refined with *U*_iso_(H) = 1.5*U*_eq_(O). The C-bound H-atoms were included in calculated positions and treated as riding atoms: C—H = 0.93, 0.96 and 0.97 Å, for CH, CH_3_ and CH_2_ H atoms, respectively, with *U*_iso_(H) = 1.5*U*_eq_(C) for methyl H atoms and = 1.2*U*_eq_(C) for other H atoms.

### Crystal data

**Table:**

C_10_H_10_N_4_O_2_S_2_	*Z* = 2
*M**_r_* = 282.34	*F*(000) = 292
Triclinic, *P*1	none
Hall symbol: -P 1	*D*_x_ = 1.489 Mg m^−^^3^
*a* = 7.1500 (3) Å	Mo *K*α radiation, λ = 0.71073 Å
*b* = 8.3770 (3) Å	Cell parameters from 2086 reflections
*c* = 11.0890 (5) Å	θ = 10.4–19.8°
α = 74.7480 (14)°	µ = 0.42 mm^−^^1^
β = 79.3090 (14)°	*T* = 290 K
γ = 86.286 (3)°	Prism, yellow
*V* = 629.58 (4) Å^3^	0.1 × 0.05 × 0.05 mm

### Data collection

**Table:**

Bruker–Nonius KappaCCD diffractometer	2335 independent reflections
Radiation source: Fine-focus	1879 reflections with *I* > 2σ(*I*)
Graphite monochromator	*R*_int_ = 0.079
CCD scans	θ_max_ = 25.7°, θ_min_ = 3.8°
Absorption correction: for a cylinder mounted on the φ axis (Dwiggins, 1975)	*h* = −8→8
*T*_min_ = 0.861, *T*_max_ = 0.862	*k* = −10→10
15888 measured reflections	*l* = −13→13

### Refinement

**Table:**

Refinement on *F*^2^	0 restraints
Least-squares matrix: full	H atoms treated by a mixture of independent and constrained refinement
*R*[*F*^2^ > 2σ(*F*^2^)] = 0.051	*w* = 1/[σ^2^(*F*_o_^2^) + (0.0968*P*)^2^ + 0.1021*P*] where *P* = (*F*_o_^2^ + 2*F*_c_^2^)/3
*wR*(*F*^2^) = 0.15	(Δ/σ)_max_ < 0.001
*S* = 1.04	Δρ_max_ = 0.50 e Å^−^^3^
2335 reflections	Δρ_min_ = −0.30 e Å^−^^3^
167 parameters	

### Special details

**Table:**

Geometry. All e.s.d.'s (except the e.s.d. in the dihedral angle between two l.s. planes) are estimated using the full covariance matrix. The cell e.s.d.'s are taken into account individuallno in the estimation of e.s.d.'s in distances, angles and torsion angles; correlations between e.s.d.'s in cell parameters are onlno used when theno are defined bno crnostal snommetrno. An approximate (isotropic) treatment of cell e.s.d.'s is used for estimating e.s.d.'s involving l.s. planes.

### Fractional atomic coordinates and isotropic or equivalent isotropic displacement parameters (Å^2^)

**Table:**

	*x*	*y*	*z*	*U*_iso_*/*U*_eq_	
S1	0.12427 (8)	0.32835 (9)	0.14822 (6)	0.0644 (3)	
S2	0.31586 (12)	0.16473 (8)	0.46904 (6)	0.0744 (3)	
O1	0.3263 (3)	0.0525 (3)	−0.07328 (19)	0.0753 (6)	
H1	0.367 (6)	−0.039 (5)	−0.072 (4)	0.113*	
O2	0.1581 (4)	−0.0338 (3)	0.1185 (2)	0.0973 (8)	
N1	0.5089 (3)	0.2791 (3)	0.0763 (2)	0.0593 (5)	
N2	0.6701 (3)	0.3173 (3)	0.1112 (2)	0.0621 (5)	
N3	0.6313 (3)	0.3917 (3)	0.2004 (2)	0.0593 (5)	
N4	0.4384 (2)	0.4047 (2)	0.22640 (17)	0.0490 (4)	
C1	0.2079 (3)	0.0747 (3)	0.0282 (2)	0.0576 (6)	
C2	0.1398 (3)	0.2518 (3)	0.0096 (2)	0.0546 (5)	
H2A	0.0153	0.2615	−0.0148	0.066*	
H2B	0.2261	0.3207	−0.0597	0.066*	
C3	0.3657 (3)	0.3352 (3)	0.1496 (2)	0.0507 (5)	
C4	0.3483 (3)	0.4803 (3)	0.3259 (2)	0.0497 (5)	
C5	0.3341 (4)	0.6503 (3)	0.2979 (2)	0.0597 (6)	
H5	0.3723	0.7138	0.2153	0.072*	
C6	0.2621 (4)	0.7248 (4)	0.3944 (3)	0.0721 (7)	
H6	0.2489	0.8394	0.3774	0.087*	
C7	0.2099 (4)	0.6277 (4)	0.5165 (3)	0.0736 (8)	
H7	0.1645	0.6782	0.5817	0.088*	
C8	0.2235 (4)	0.4586 (4)	0.5438 (2)	0.0637 (6)	
H8	0.1866	0.396	0.6267	0.076*	
C9	0.2925 (3)	0.3800 (3)	0.4476 (2)	0.0539 (5)	
C10	0.2427 (5)	0.0824 (4)	0.6350 (3)	0.0923 (10)	
H10A	0.3274	0.1185	0.68	0.138*	
H10B	0.2456	−0.0363	0.6544	0.138*	
H10C	0.1156	0.1204	0.6604	0.138*	

### Atomic displacement parameters (Å^2^)

**Table:**

	*U*^11^	*U*^22^	*U*^33^	*U*^12^	*U*^13^	*U*^23^
S1	0.0435 (4)	0.0871 (5)	0.0716 (5)	−0.0001 (3)	−0.0027 (3)	−0.0413 (4)
S2	0.0991 (6)	0.0597 (4)	0.0580 (4)	−0.0016 (3)	−0.0056 (3)	−0.0094 (3)
O1	0.0897 (14)	0.0694 (12)	0.0682 (12)	0.0092 (10)	−0.0035 (10)	−0.0296 (10)
O2	0.1136 (18)	0.0716 (13)	0.0805 (14)	0.0116 (12)	0.0131 (12)	0.0020 (11)
N1	0.0483 (10)	0.0683 (12)	0.0648 (12)	−0.0004 (9)	0.0007 (8)	−0.0307 (10)
N2	0.0473 (10)	0.0726 (13)	0.0670 (13)	0.0032 (9)	−0.0015 (9)	−0.0260 (11)
N3	0.0434 (10)	0.0711 (13)	0.0628 (12)	0.0002 (8)	−0.0052 (8)	−0.0192 (10)
N4	0.0425 (9)	0.0543 (10)	0.0501 (10)	0.0014 (7)	−0.0045 (7)	−0.0163 (8)
C1	0.0544 (12)	0.0648 (14)	0.0558 (14)	−0.0015 (10)	−0.0109 (10)	−0.0186 (11)
C2	0.0496 (12)	0.0600 (13)	0.0571 (13)	−0.0012 (10)	−0.0111 (10)	−0.0188 (11)
C3	0.0475 (11)	0.0545 (12)	0.0522 (12)	0.0008 (9)	−0.0048 (9)	−0.0205 (10)
C4	0.0460 (11)	0.0569 (12)	0.0506 (12)	0.0024 (9)	−0.0103 (9)	−0.0206 (10)
C5	0.0611 (14)	0.0563 (14)	0.0640 (14)	0.0032 (10)	−0.0140 (11)	−0.0184 (11)
C6	0.0711 (16)	0.0646 (16)	0.094 (2)	0.0088 (12)	−0.0231 (14)	−0.0392 (15)
C7	0.0624 (15)	0.095 (2)	0.0812 (19)	0.0070 (14)	−0.0154 (13)	−0.0530 (17)
C8	0.0594 (14)	0.0837 (18)	0.0538 (13)	0.0016 (12)	−0.0090 (10)	−0.0289 (12)
C9	0.0481 (11)	0.0649 (14)	0.0514 (12)	0.0001 (10)	−0.0096 (9)	−0.0193 (10)
C10	0.096 (2)	0.093 (2)	0.0671 (18)	0.0006 (17)	−0.0007 (15)	0.0068 (16)

### Geometric parameters (Å, º)

**Table:**

S1—C3	1.734 (2)	C2—H2B	0.97
S1—C2	1.798 (2)	C4—C5	1.376 (3)
S2—C9	1.757 (3)	C4—C9	1.391 (3)
S2—C10	1.778 (3)	C5—C6	1.381 (4)
O1—C1	1.324 (3)	C5—H5	0.93
O1—H1	0.80 (4)	C6—C7	1.380 (4)
O2—C1	1.177 (3)	C6—H6	0.93
N1—C3	1.327 (3)	C7—C8	1.369 (4)
N1—N2	1.364 (3)	C7—H7	0.93
N2—N3	1.282 (3)	C8—C9	1.395 (3)
N3—N4	1.359 (3)	C8—H8	0.93
N4—C3	1.341 (3)	C10—H10A	0.96
N4—C4	1.444 (3)	C10—H10B	0.96
C1—C2	1.504 (3)	C10—H10C	0.96
C2—H2A	0.97		
			
C3—S1—C2	98.45 (10)	C9—C4—N4	118.91 (19)
C9—S2—C10	104.17 (14)	C4—C5—C6	118.8 (2)
C1—O1—H1	118 (3)	C4—C5—H5	120.6
C3—N1—N2	105.52 (19)	C6—C5—H5	120.6
N3—N2—N1	111.51 (18)	C7—C6—C5	119.3 (3)
N2—N3—N4	106.22 (18)	C7—C6—H6	120.3
C3—N4—N3	108.48 (17)	C5—C6—H6	120.3
C3—N4—C4	131.62 (18)	C8—C7—C6	121.6 (2)
N3—N4—C4	119.88 (18)	C8—C7—H7	119.2
O2—C1—O1	123.2 (2)	C6—C7—H7	119.2
O2—C1—C2	125.3 (2)	C7—C8—C9	120.2 (2)
O1—C1—C2	111.4 (2)	C7—C8—H8	119.9
C1—C2—S1	113.73 (17)	C9—C8—H8	119.9
C1—C2—H2A	108.8	C4—C9—C8	117.2 (2)
S1—C2—H2A	108.8	C4—C9—S2	117.76 (17)
C1—C2—H2B	108.8	C8—C9—S2	125.01 (19)
S1—C2—H2B	108.8	S2—C10—H10A	109.5
H2A—C2—H2B	107.7	S2—C10—H10B	109.5
N1—C3—N4	108.27 (19)	H10A—C10—H10B	109.5
N1—C3—S1	127.70 (17)	S2—C10—H10C	109.5
N4—C3—S1	124.01 (16)	H10A—C10—H10C	109.5
C5—C4—C9	122.8 (2)	H10B—C10—H10C	109.5
C5—C4—N4	118.2 (2)		

### Hydrogen-bond geometry (Å, º)

**Table:**

*D*—H···*A*	*D*—H	H···*A*	*D*···*A*	*D*—H···*A*
O1—H1···N1^i^	0.81 (4)	2.15 (4)	2.952 (4)	176 (4)
O1—H1···N2^i^	0.81 (4)	2.51 (4)	3.232 (4)	149 (4)

Symmetry code: (i) −*x*+1, −*y*, −*z*.
